# Prediction of anastomotic insufficiency based on the mucosal microbiome prior to colorectal surgery: a proof-of-principle study

**DOI:** 10.1038/s41598-024-65320-w

**Published:** 2024-07-03

**Authors:** Konrad Lehr, Undine Gabriele Lange, Noam Mathias Hipler, Ramiro Vilchez-Vargas, Albrecht Hoffmeister, Jürgen Feisthammel, Dorina Buchloh, Denny Schanze, Martin Zenker, Ines Gockel, Alexander Link, Boris Jansen-Winkeln

**Affiliations:** 1 Department of Gastroenterology, Hepatology and Infectious Diseases, Faculty of Medicine, Section of Molecular Gastroenterology and Microbiota-Associated Diseases, Otto Von Guericke University Magdeburg, Magdeburg, Germany; 2 Clinic and Polyclinic for Visceral, Transplant, Thoracic and Vascular Surgery, Faculty of Medicine, University of Leipzig, Leipzig, Germany; 3 Clinic and Polyclinic for Oncology, Gastroenterology, Hepatology and Pneumology, Faculty of Medicine, University of Leipzig, Leipzig, Germany; 4 Clinic for General and Visceral Surgery, Protestant Deaconess House Leipzig, Leipzig, Germany; 5 Institute of Human Genetics, Faculty of Medicine, Otto Von Guericke University Magdeburg, Magdeburg, Germany; 6 Clinic for General, Visceral, Thoracic and Vascular Surgery, Clinic St. Georg Leipzig, Leipzig, Germany

**Keywords:** Gastroenterology, Surgical oncology, Predictive markers, Translational research

## Abstract

Anastomotic leakage (AL) is a potentially life-threatening complication following colorectal cancer (CRC) resection. In this study, we aimed to unravel longitudinal changes in microbial structure before, during, and after surgery and to determine if microbial alterations may be predictive for risk assessment between sufficient anastomotic healing (AS) and AL prior surgery. We analysed the microbiota of 134 colon mucosal biopsies with 16S rRNA V1-V2 gene sequencing. Samples were collected from three location sites before, during, and after surgery, and patients received antibiotics after the initial collection and during surgery. The microbial structure showed dynamic surgery-related changes at different time points. Overall bacterial diversity and the abundance of some genera such as *Faecalibacterium* or *Alistipes* decreased over time, while the genera *Enterococcus* and *Escherichia_Shigella* increased. The distribution of taxa between AS and AL revealed significant differences in the abundance of genera such as *Prevotella*, *Faecalibacterium* and *Phocaeicola*. In addition to *Phocaeicola*, *Ruminococcus2* and *Blautia* showed significant differences in abundance between preoperative sample types. ROC analysis of the predictive value of these genera for AL revealed an AUC of 0.802 (p = 0.0013). In summary, microbial composition was associated with postoperative outcomes, and the abundance of certain genera may be predictive of postoperative complications.

## Introduction

Colorectal cancer (CRC) is one of the most common cancers in both men and women and is the fourth deadliest tumour^1^. The incidence of CRC is particularly high in western industrialized nations, since factors such as obesity, processed meat and type II diabetes mellitus favour the development of CRC^2–4^. Despite the established knowledge of the risk factors, an increasing incidence is predicted for many European countries in the coming years^1^. Furthermore, due to the good preventive and screening options, there is the possibility of an earlier diagnosis^5^, which enables curative therapy, which always includes endoscopic or surgical removal of the tumour, for example by low anterior resection, and supplemented chemotherapy depending on the stage^6^. Despite novel options for curative treatment using immunotherapy for a subset of patients, the surgical resection remains the key stone in the standard of care. With adequate distance to the sphincter, continuity of the bowel can be restored after oncologic resection by means of an anastomosis. After rectal resection, a protective stoma is often created to protect the anastomosis. Nevertheless, despite technical advances over the past years, an anastomotic leakage (AL) remains in 2–19% and can cause potentially life-threatening complications^7^. Some patient-related and therapy-related risk factors have been identified, such as obesity or neoadjuvant therapies^8^.

Although genetic factors may potentially be associated with AL the microbial composition is increasingly considered as one of the key features defining health and disease and potentially be involved in healing process. Since aging and environment are the key elements that impact microbial dynamics, the precise knowledge may offer a diagnostic and therapeutic opportunities^9^. Even though there is no common definition of the “healthy” microbiome, several bacterial species like *Phocaeicola*, *Bacteroides*, *Prevotella*, *Alistipes*, *Ruminococcus* and *Faecalibacterium* and others as well as bacterial diversity are associated with healthy gut^9–11^. Furthermore, a lot of data has been collected on the microbiome profile of various diseases and it is believed to play a role in the prevention, prediction, diagnosis and further therapy^12,13^. In terms of CRC, *Fusobacterium nucleatum* (*F. nucleatum)* has been linked to colon carcinogenesis as it is enriched in the CRC primary tumour or metastatic tissues and is associated with CRC progression, recurrence, and with worse prognosis in CRC^14,15^. Recently, it has been suggested that the microbiota may plays not only an important role in CRC but furthermore in the occurrence of AL. Different aspects of the relationship of AL and the microbiota have been discussed like the influences of the intestinal mucus layer, pre-operative manipulations of the microbiota or the impact of peri-operative interventions^16,17^. Overall, the data strongly suggest that the microbiome contributes to anastomotic healing in addition to surgical technique or, conversely, that the relationship between host and microbiota may play a role in causing and/or exacerbating AL. However, the extent to which the location of the harvested biopsies specimens or the longitudinal microbial structure before, during, and after surgery have an influence is still unclear. Identifying potentially modifiable factors, such as microbial environment, that may predict the risk of developing complications may provide an opportunity for prevention and improved patient management.

**Figure 1:**
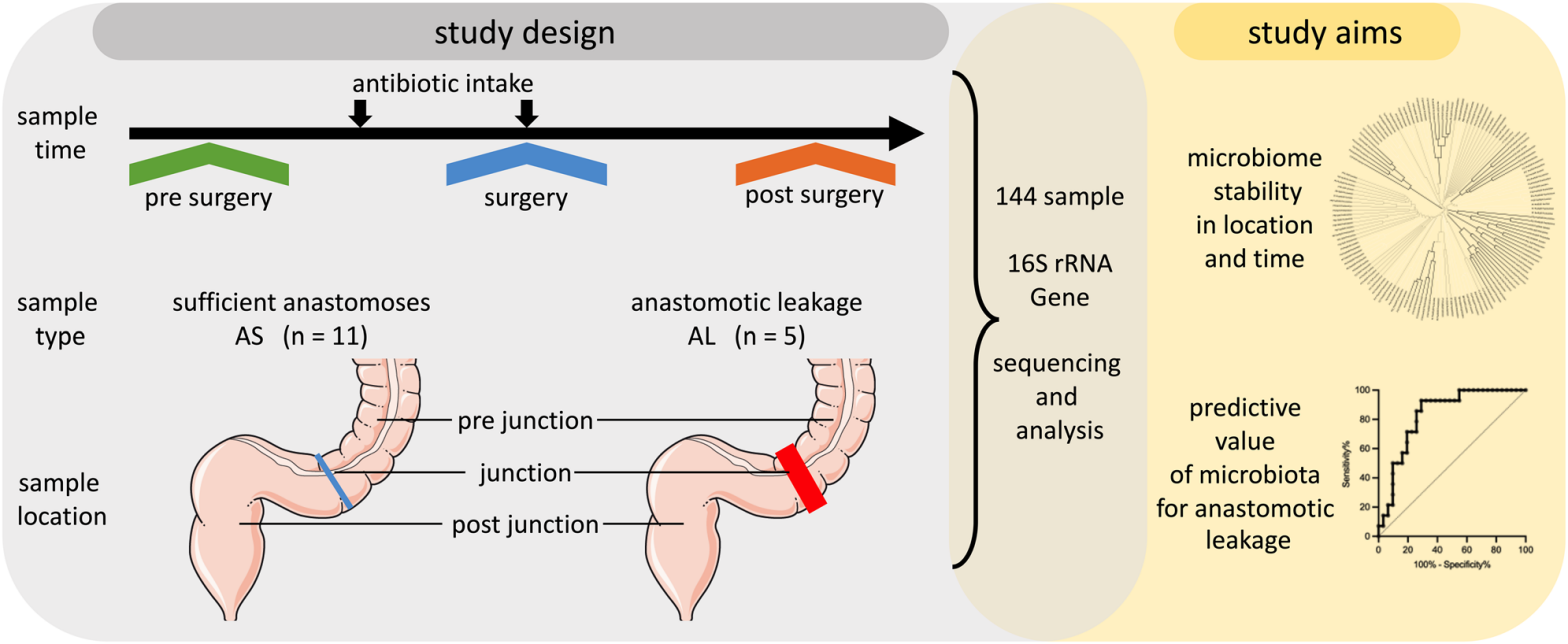
Graphical abstract of the study.

Therefore, this study aimed to systematically evaluate the mucosal microbial alterations in subjects undergoing tumour resection and identify potential microbial markers in the lower gastrointestinal bacterial community that could have predictive value for AL (Fig. 1).

## Results

Following sequencing, filtering, merging and normalization the bacterial community of biopsies sample was annotated to 4114 unique phylotypes belonging to 259 genera with Phocaeicola (9%), Bacteroides (7%) and Enterococcus (6%) as the most abundant ones (Supplementary Table 1 and 2).

### Locational stability in global bacterial community

**Figure 2:**
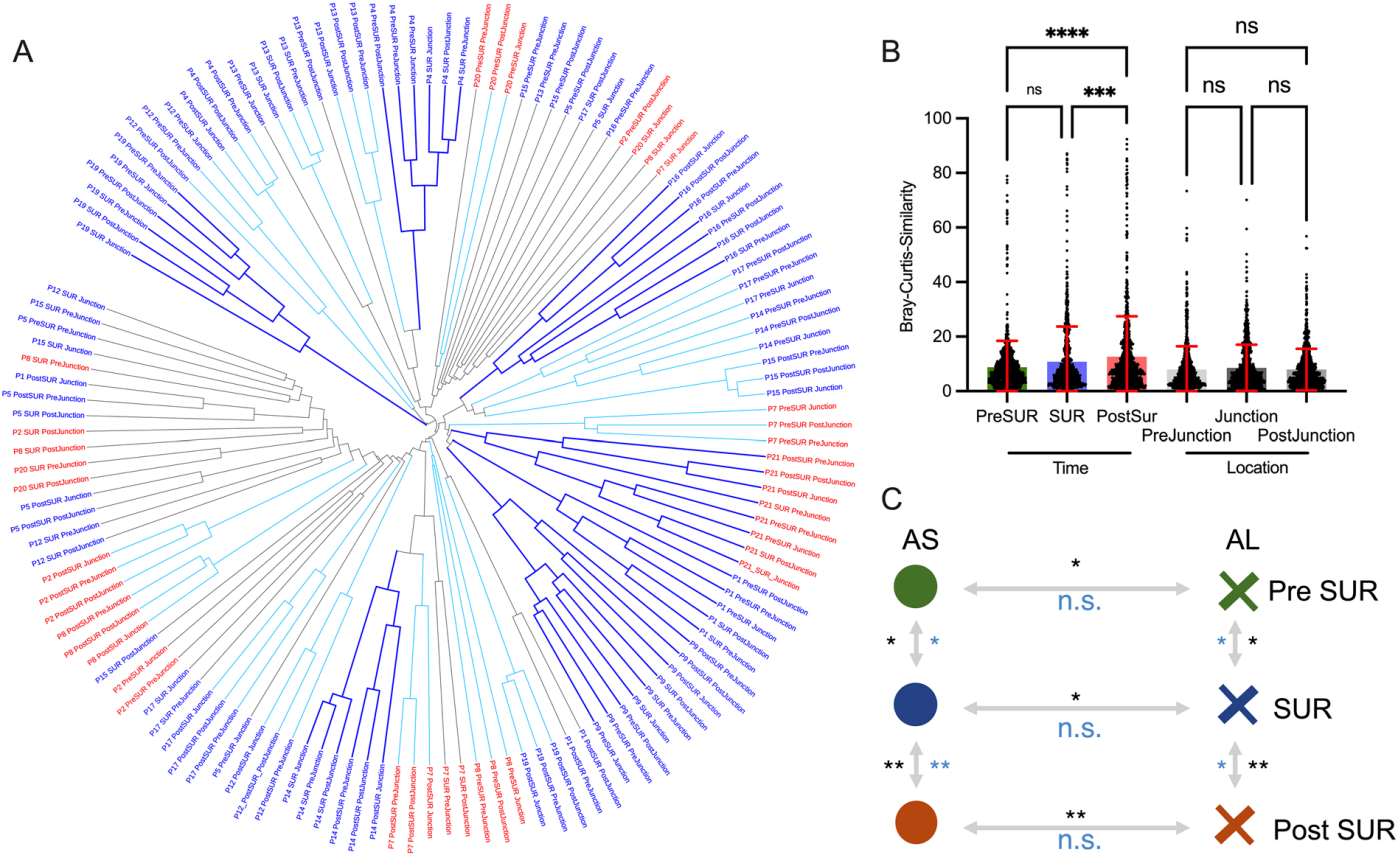
Microbial stability at different locations and time points. (**A**) Dendrogram of all sample underlying a Bray–Curtis resemblance measurement at Phylotype level. Sample of AS and AL displayed in blue and red, respectively. (**B**) Paired comparison of Bray–Curtis-Similarity of different groups. (**C**) Multivariate analysis between different groups underlying a Bray–Curtis resemblance measurement at genus level (ANOSIM displayed in blue, PERMANOVA displayed in black).

The bacterial community profiles of the whole cohort were compared for their Bray–Curtis-similarity on phylotype level with hierarchical clustering (Fig. 2A). Sample from the same patient at same timepoint clustered typically together (66%) similar as previously described^11,18^, suggesting a comparable microbial community in these sample and an overall low locational intraindividual variability. In 6 cases the microbiota clustered together and remained stable over time even after the antibiotic intake. All possible combinations for the paired Bray–Curtis-similarity of two sample were analysed for differences, dividing them by sampling time and location factor (Fig. 2B). When sorted for the timepoints (PreSUR, SUR and PostSUR) the Bray–Curtis similarity showed significant differences between all timepoints, with the lowest value before the surgery, suggesting a higher variability at this timepoint. In contrast to that, if the sample were sorted for the sample locations (PreJunction, Junction and PostJunction) no differences could be detected. Furthermore, multivariate analysis with ANOSIM and PERMANOVA could only find statistical differences between timepoints and the anastomotic phenotype (AS and AL). Nevertheless, due to the substantial variation among the AS and AL the groups could not be clearly separated and ANOSIM failed to reach the level of significance (Fig. 2C). No significant difference was found between the sample locations (PreJunction, Junction and PostJunction) even with multivariate tests (PERMANOVA p-value = 0.998, ANOSIM p-value = 0.997, data not shown).

### Postsurgical decrease in bacterial diversity

**Figure 3:**
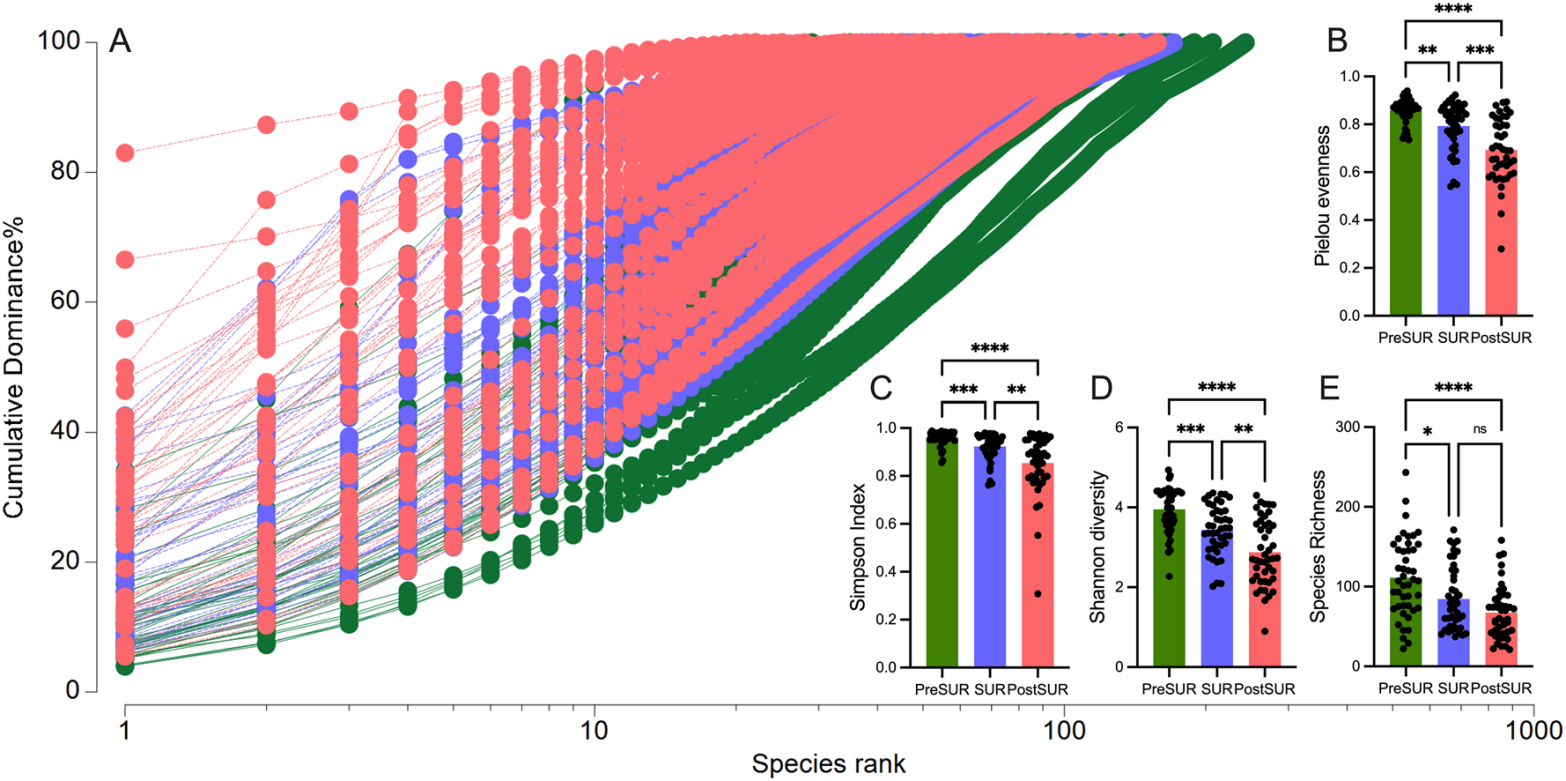
Reduced microbial diversity following surgery. (**A**) Dominance plot for all sample classified for their timepoint. Comparison of diversity measurements (**B**) Pilous evenness, (**C**) Simpson Index, (**D**) Shannon diversity and (**E**) Species richness.

The surgical procedure and concomitant periinterventional antibiotic treatment had as expected strong effect on the diversity of the bacterial community. As shown in the dominance plot (Fig. 3A), the SUR and PostSUR samples were dominated by a single species in comparison to PreSUR specimens. Furthermore, all applied diversity measurements decreased significantly over time (Pielou’s evenness, Simpson Index, Shannon diversity, Species Richness; Fig. 3B–E). However, there was no significant difference for diversity of sample belonging to AS or AL groups (Supplementary Fig. 1). Surprisingly, when the diversity of the sample after surgery (PostSUR) with and without stoma were compared, only the Pilous evenness showed a significant difference. In addition, the bacterial community in the group without stoma was more evenly distributed than the sample group with a stoma (Supplementary Fig. 1).

### Differences in microbial structure in patients with and without AL

**Figure 4:**
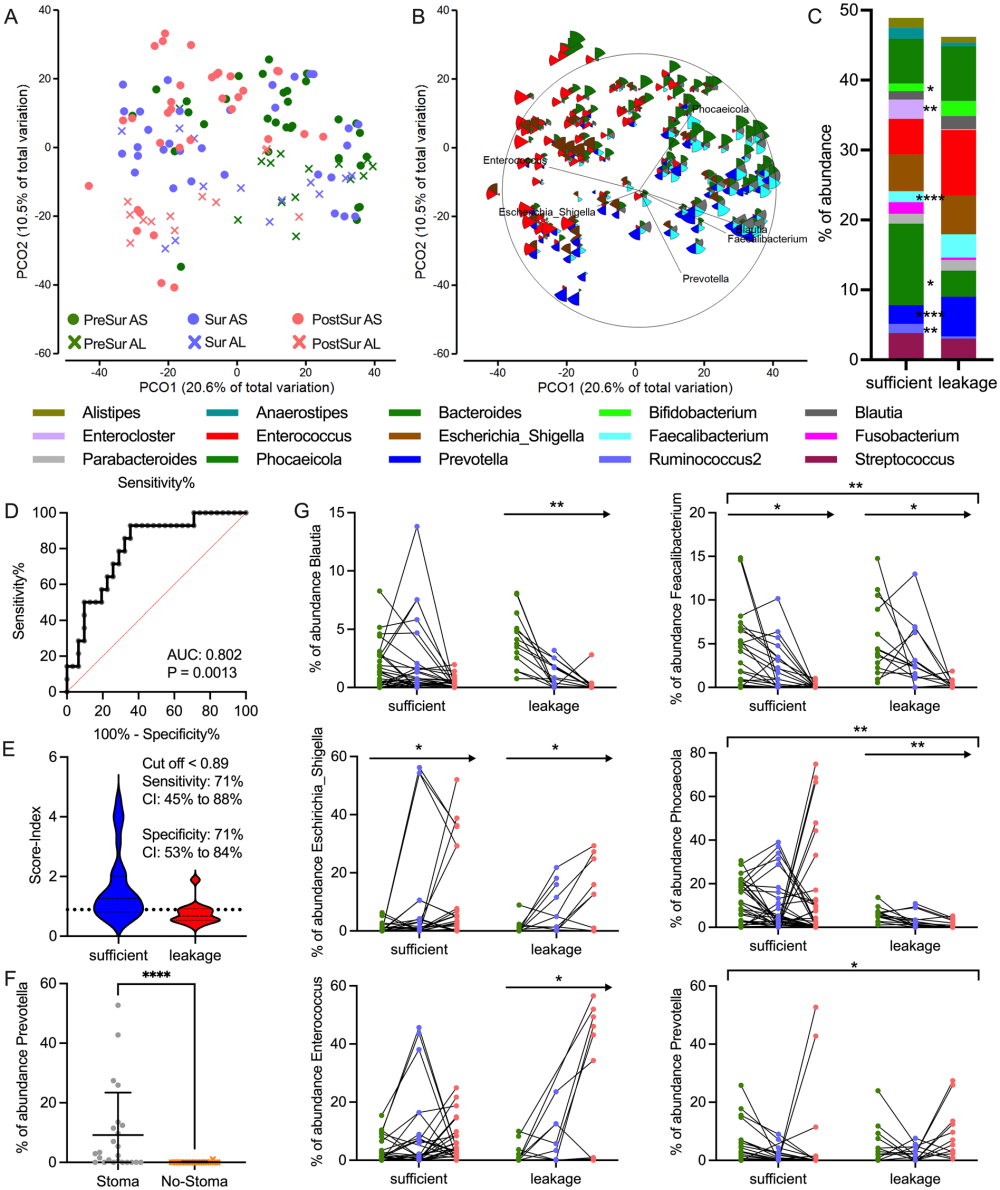
Microbial differences between patients with and without AL. PCO underlying a Bray–Curtis resemblance measurement at genus level displaying groups (**A**) and with bubble-plot and vector overlay representing the abundance of genera across the sample (**B**). (**C**) Comparison of the average relative abundance of bacteria in AS and AL across all timepoints. (**D**) ROC-analysis for the occurrence of AL based on the abundance of certain bacterial genera. (**E**) Violin-plot of the score-index applied in the ROC-analysis, with the best suited cut-off displayed as dashed line (95% CI). (**F**) Comparison of the genus *Prevotella* between patients with and without stoma. (**G**) Timepoint specific relative abundance of bacteria in AS and AL. Arrow displays statistically significant trend over time in one group (trendyspliner-Test). Bracket displays statistically significant differences between two groups over the full-time course (permuspliner-Test).

The clinical characteristics of the patients with and without AL were evaluated and no significant differences were found, in sex, tumour localisation, histological classification, nicotine abuse or diabetes (data not shown). However, since the multivariate analysis of Fig. 2C indicated the structural differences in the microbiome of AS and AL samples, we performed further in-depth analysis. A PCO analysis revealed different clustering of AS- and AL-sample, which was supported by applying the Bootstrap-average method (Fig. 4A, Supplementary Fig. 2). The microbial structure was considerable different for the sample types with *Prevotella* more common in the AL-cohort, *Phocaeicola* in the AS-sample and *Enterococcus* mostly abundant in sample after surgery (PostSUR). The PCO suggests also an antagonistic relationship between those bacteria, which further reinforces the different structure of the microbiota (Fig. 4B). Analysis of the distance among the centroid for the different sample locations reveals similar results than the PCO with all sample, strengthen the fact that the differences between sample locations are neglectable. With these results the trend to more *Enterococcus* after the surgery is clearer, while main differences between AS- and AL sample seems to be stable over time (Supplementary Fig. 2).

Analysis of the taxa distribution between the groups showed statistical difference in the abundance of 6 different genera between AS and AL-groups, specially of *Prevotella* and *Faecalibacterium* (q < 0.0001) with a greater abundance in AL-sample and *Phocaeicola* and *Ruminococcus2* with a significant higher abundance in AS-sample. Some of these differences were even significant when the timepoints were analysed separately (Fig. 4C, Supplementary Fig. 2). *Phocaeicola* and *Ruminococcus2* were significantly more abundant in the AS-sample before the surgery, while *Blautia* and *Faecalibacterium* were more abundant in the AL-group at the PreSUR timepoint (Supplementary Fig. 2). To determine the predictive value of these genera for AL a ROC-analysis was performed and the resulting score showed an area under the curve (AUC) of 0.802 (p = 0.0013) and could effectively predict AL on the basis of pristine preoperative microbiota (Fig. 4D). The risk for AL was highest with a score-index below 0.89 with a sensitivity of 71% (95% CI 45–88%) and a specificity of 71% (95% CI 53–84%, Fig. 4E).

Postoperative specimens were examined to determine whether the microbiome was affected by a stoma. The taxa distribution revealed that *Prevotella* was highly more abundant in patients with a stoma (q < 0.0001) and *Phocaeicola* among others was more abundant in patients where without stoma (Fig. 4F, Supplementary Fig. 3).

Finally, longitudinal analysis of taxa distribution was extended using the R package splinectomeR, which is specifically designed for this type of analysis. A significant trend over time was found for several taxa, mostly in the AL-sample. Abundance of *Alistipes, Blautia, Phocaeicola* and *Faecalibacterium* decreased over time while on the other hand the abundance of *Enterococcus* and *Escherichia_Shigella* increased. For *Phocaeicola, Prevotella, Anaerostipes, Faecalibacterium* and *Ruminococcus2* statistically significant abundance differences were found between the two groups during the full-time course (Fig. 4G, Supplementary Fig. 4).

### Bacterial species profile of the most abundant genera

**Figure 5:**
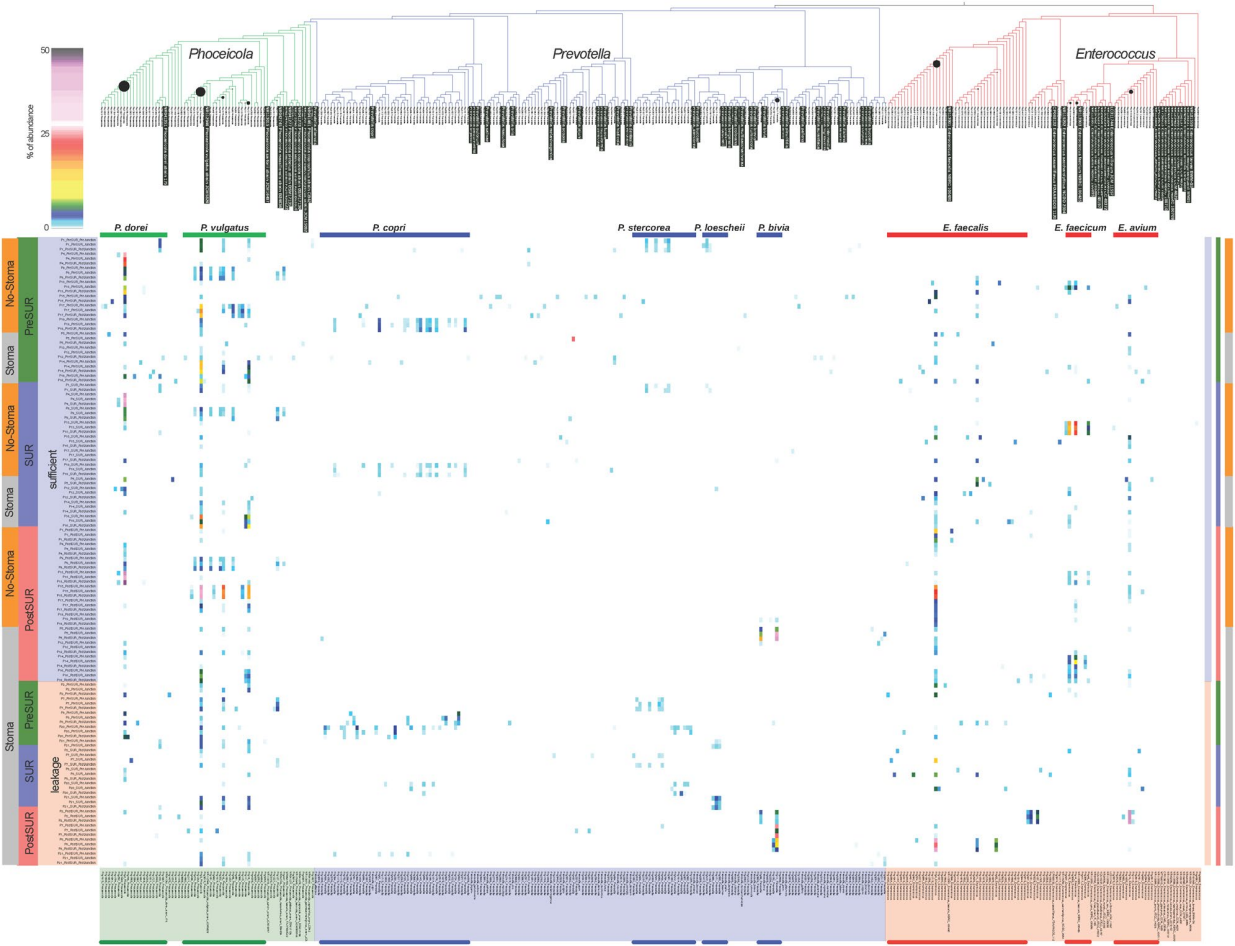
Phylotype distribution of *Enterococcus, Prevotella* and *Phocaeicola*. Type strains from the platform NCBI^26^ shown with black background. The abundance of the phylotypes is displayed by the diameter of the black circles. Phylotype belonging to abundant species are labeled. Phylotype in heatmap follow the dendrogram order.

Further, the sequences of the V1-V2 region of the 16S rRNA gene from 274 bacterial phylotypes detected in the cohort and belonging to the most abundant genera *Phocaeicola*, *Prevotella* and *Enterococcus* were compared with all type strains of these genera to define the discriminatory power of each sequence read (Fig. 5), similar as described previously^19^. The most abundant genus in the cohort was *Phocaeicola* with the dominant species *P. dorei* (Phy2, Average-abundance: 3.3%, Max-abundance: 46%) and *P. vulgatus* (Phy3, AVG: 2.8%, Max: 36%). High abundances (above 5%) of these phylotype and other phylotype belonging to the two species were distributed throughout the cohort, but were only present in the patient group with sufficient anastomoses. *Prevotella* had the greatest phylotype diversity of the analyzed genera, but the different phylotypes were not very common in the cohort, the most abundant one is present in only 16 sample. A wide range of phylotypes belonging to *P. copri* was present in samples before the surgery (PreSUR) and in samples taken during the surgery (SUR) in a lower abundance. After the surgery and the second dose on antibiotics the phylotype profile of *Prevotella* phylotype was far less divers, dominated by only two phylotypes of *P. bivia* (Phy11, AVG: 1.3%, Max: 37% and Phy66, AVG: 0.2%, Max: 15%). The third genus, *Enterococcus*, consist mainly of three species *E. faecalis* (Phy4, AVG: 2.2%, Max: 33%), *E. faecium* (Phy14, AVG: 0.8%, Max: 23% and Phy19, AVG: 0.5%, Max: 15%) and *E. avium* (Phy15, AVG: 1.2%, Max: 42%). These phylotype were only present in high abundances in samples during (SUR) and after the surgery (PostSUR), after one or two doses of antibiotics, suggesting a possible resistance of these bacteria. In the heatmap most of the described phylotype were present in three subsequent sample, displaying the local microbial stability already globally observed in Fig. 2A.

## Discussion

Changes in the microbiome following colorectal cancer surgery have been previously reported, but little is known on the microbial dynamics throughout different steps in surgical management. In this study, we systematically analysed the mucosal microbiome of colorectal tissues along the different sampling sites and periinterventional time points and evaluated the potential of microbiome to predict AL based on microbial profile prior surgery. Our data showed a distinct change in the microbiome over time with a significant loss of diversity but also changes in the abundance of bacterial genera. The significant differences in abundance of the genera *Phocaeicola*, *Ruminococcus2*, *Faecalibacterium* and *Blautia* between AS and AL suggested its potential as a predictive biomarker for AL.

The gut microbiota has by far the greatest diversity of microbial communities in the human body and is a very complex and still not fully understood ecosystem. Yet the microbial variation of sample from approximate same locations in the gut is quite low, which has been confirm with our data^11^. In particular, at phylotype level the shared abundances of the sequences belonging to the most abundant genera (*Phocaeicola, Prevotella* and *Enterococcus*) contributed to the similarity of sampling sites. Similar trend has been also reported for the microbiome of the gastric mucosa^18^. For comparison, microbiome analysis of the surgical specimens of the stomach showed slightly greater difference^19^. In the present cohort, due to the study design we applied different modalities including endoscopy-assisted biopsy collection and surgical specimens’ assessment during surgery to minimize potential sampling bias.

To our knowledge, longitudinal changes in the microbiome during surgical resection has not been analysed as systematically and in detail as in our study. First, we observed several differences between time points, implying that surgical procedure and most importantly antibiotic treatment had a clear overall effect between sampling time points. We observed the loss of species diversity in the samples and a decreasing abundance of many genera frequently linked with a “healthy state”, but also an increasing abundance of genera such as *Enterococcus* and *Escherichia_Shigella*. Regarding the significant impact of surgical procedure on the microbiome of intestinal tract have already reported previously. Ohigashi et al. demonstrated a postoperative decrease in obligate anaerobes and an increase in populations of certain bacteria such as *Enterobacteriaceae, Enterococcus, Staphylococcus*, and *Pseudomonas*^20^. *E. faecalis* has also been associated with AL in the literature. Lee et al. performed a retrospective study to examine the pathogens isolated from blood or peritoneal fluid in patients who developed AL after CRC surgery and reported that *E. faecalis* was among the most common pathogens isolated from leak sites^21^, which is confirmed in our study. The increase in *Enterococcus* species, including *E. faecalis*, may be likely due to the antibiotic resistances in this genus as was recently demonstrated in a whole-genome sequencing study^22^. The effect of antibiotics was also measurable at the phylotype level where *P. copri* was almost completely replaced by *P. bivia* after two courses of antibiotics. Since *P. copri* is a common and widely distributed habitant in the gut microbiota^23^, these changes may have substantial impact on the bacterial network. This becomes even more important as microbial alteration such as reduced species diversity and an increase in antibiotic-resistant bacterial strains following antibiotic exposure can last up to 2 years^24^.

The main question of the study was to evaluate if mucosal microbial pattern may be predictive for AL prior surgery. Indeed, the data from this study identified several structural differences between patients with AS and AL, where some of those were even quite stable across different sampling time points. For example, *Phocaeicola* was found to be associated with AS, whereas *Prevotella* was associated with AL. It has to be mentioned that previous studies reported on opposite results^25^, therefore further studies are necessary here, but the involvement of these two genera is very intriguing because *Phocaeicola*, previously classified as *Bacteroides*^26^ , and *Prevotella* are the two most important and abundant bacteria in the lower gastrointestinal tract^9–11^. These genera also show a strong antagonistic relationship^27,28^, which further strengthens the microbial structure of the AS and AL sample and could be important for modifying the microbiota of the colorectal cancer environment, e.g., with pro- or prebiotics or faecal microbiome transplantation.

Increased abundance of *Enterococcus* has been reported in the past in patients who developed AL after CRC surgery^21^. Komen et al. evaluated the presence of *E.* *faecalis* in intra-abdominal drains after colon surgery using reverse transcription polymerase chain reaction (RT-PCR) and suggest that the presence of these bacteria postoperatively may represent a strategy to detect AL^29^. Although Enterococcus was indeed linked to AL, it was not sufficient for prediction of AL development. The microbial pattern specifically prior surgery identified *Phocaeicola*, *Ruminococcus2*, *Faecalibacterium* and *Blautia* with a potential to predict the course of the anastomotic healing. Nevertheless, despite the remarkable predictive value for this relatively small cohort, the score may be further optimized by other known risk factors, especially low preoperative haemoglobin, contamination of the operative field and hyperglycaemia in the future^30^. Furthermore, combination of the pre and post microbial assessment could potentially be further considered for assessment of AL.

Given the novelty of the data, there are also few limitations of the study. The quite intense study protocol and a corona pandemic restriction allowed to include only a limited patient number for this proof-of-principle study. Therefore, larger studies with systematic presurgical samples collection are needed to confirm our results. Furthermore, in this conceptual work, we clearly focused on microbial changes. However, parallel assessments of bacterial cultures, metabolites, or functional (protein) changes could provide additional insights into causality and functional roles. With this data, there is potential to implement this model in vitro to explore therapeutic options. In addition, patients were recruited from a single center. Samples from a multicenter study would not only help mitigate potential environmental bias but also allow for the assessment of differences in leakage frequency and subsequent risk factors.

In conclusion, systematic microbial analysis of the mucosa from patients with and without AL revealed bacterial differences that may be of potential translation value in the pathogenesis of AL. Assessment of bacterial alteration prior surgery may serve as a predictive tool of AL development. We clearly confirm that the antibiotic exposure leads to significant alterations of bacterial diversity and selective growth of Enterococcus and other bacteria. The data of the study clearly underline an emerging role of microbiome in surgical complication and suggest the potential of microbial intervention in prevention and treatment of AL.

## Material and methods

### Study cohort and sample collection

The study cohort consisted of 4 colon- and 12 rectal cancer patients including 12 men and 4 women with a mean age of 60 ± 8 years, who underwent low anterior resection of the rectum with following anastomoses. 8 patients received neoadjuvant therapy, 11 patients showed no evidence for anastomotic leakage (AS) and 5 with AL (Supplementary Fig. 5). Sample collection was performed at the Clinic and Polyclinic for Visceral, Transplantation, Thoracic and Vascular Surgery of the University Leipzig (Sachsen, Germany). The study was conducted in accordance with the current Good Clinical Practice guidelines and the Declaration of Helsinki^31^ and approved by the Ethics Committee at the Faculty of Medicine, University of Leipzig (253/19-ek). All patients gave their written informed consent to the participation in the study and were registered in clinicaltrials.org (NCT03759886). The consecutive patients were include with prior selection and the main focus was on the availability of the samples material for the analysis. The biopsy specimens were taken either during endoscopy before (PreSUR, average 4 days before) and after surgery (PostSUR, average 7 days after) or during surgery as a surgical tissue (SUR). At those timepoints three sample were taken at different location including distal the tumour (PreJunction), at the tumour or subsequently anastomosis (Junction) and proximal the tumour (PostJunction, Supplementary Fig. 5). All 144 sample were frozen in liquid nitrogen and stored under -80°C upon further analysis. Preoperative (between PreSUR and SUR) all patents received Metronidazole (1g) and Paromomycin (4g) as an oral intake and a single-shot with Ertapenem (1g) intravenous during the surgery, according to the established internal protocol (Fig. 1). Surgery was performed in a standardized manner by a single experienced surgeon. All anastomoses were tension-free, and all relevant standards, including initial anastomotic assessment (air test) and blood flow measurements (hyperspectral imaging, intraoperative indocyanine green fluorescence, and the cold steel test), showed no abnormalities or intraoperative complications. A protective ileostomy was performed according to clinical standards in patients with rectal cancer, specifically those in the lower third.

### DNA extraction and sequencing

Subsequent analysis was performed at the Otto-von-Guericke University in Magdeburg (Saxony-Anhalt, Germany). DNA was extracted from frozen biopsies samples as previously described^32^, in the first step, samples were suspended in 1 mL of lysis buffer, composed of 100 mM Tris–HCl pH 8.0, 100 mM EDTA, 100 mM NaCl, 1% (w/v) polyvinylpyrrolidone and 2% (w/v) sodium dodecyl sulphate in a 2 mL Lysing Matrix E tube (Qbiogene, Alexis Biochemicals, Carlsbad, USA). Mechanical lysis was performed with a FastPrep-24 Instrument (MP Biomedicals, Santa Ana, USA) for 40 s and 6.0 m s^−1^. The DNA extraction was followed based on phenol/chloroform as previously described^32^ and the quality and quantity of the DNA samples were determined using 1% agarose gels, and a spectrophotometric analysis of the A260/A280 ratios was also performed. Amplicon libraries were generated by amplifying the V1-V2 region of the 16S rRNA gene after 40 cycles PCR reaction with the 27F and 338R primers^33^ and paired-end sequencing on a MiSeq (2 × 300 bp; Illumina, San Diego, USA)^34^.

### Statistical analysis

All fastQ files, generated after sequencing and demultiplexing, were analysed in R Statistical Software version 4.2.1 (2022; R Foundation for Statistical Computing, Vienna, Austria) using the dada2 package (version 1.24.0)^35^, with the following standard filtering parameters. Forward and revers reads were trimmed to 240 nucleotides, the maximum of excepted Ns in the sequence was set to zero and the number of excepted errors to two, later chimeres were also removed. On average, 41% of all sequenced passed the filtering process. Resulting in a unique table containing all samples with the sequence reads (Phylotype) and their abundance. Samples were resampled to equal the smallest library size of 1064 reads using the phyloseq package (version 1.40.0)^36^ resulting in a cohort of 134 sample. Sequence reads were taxonomical annotated with the ribosomal database project^37^, based on the naïve Bayesian classification^38^ with a pseudo-bootstrap threshold of 80%. Microbial community were analysed at the taxonomic rank of phylotype and genus in relative abundances (expressed as percentages, Supplementary Table 1 and 2).

The Dominance Plot was generated with Primer 7 with PERMANOVA + add-on package (PRIMER-E, Auckland, New Zealand) based on phylotype data. PCO clustering, multivariate tests (ANOSIM^39^ and PERMANOVA^40^) and Bootstrap Average MDS^41^ were performed based on a Bray–Curtis resemblance measurement at the taxonomic rank of genus, also in Primer 7. Differences in the distribution of genera between sample types were calculated by the Mann–Whitney U unpaired test with 95% confidence interval, by using the R package exactRankTests (version 0.8–35)^42^. The resulting p values were corrected by applying the Benjamini–Hochberg false-discovery rate correction (desired FDR = 5%). The R package splinectomeR (version 0.1.0)^43,44^ was applied for longitudinal analysis of the microbiome data (trendyspliner- and permuspliner-Test) and the vegan package (version 2.6–2)^45^ for calculating diversity measurements. The Dendrogram was calculated with Bray–Curtis-similarity in R using the ecodist (version 2.0.9)^46^ and cluster (version 2.1.3)^47^ package with phylotype data as input variables and the phylogenetic tree was calculated in Seaview^48^ (clustalo alignment, distant method with 1000 bootstraps), both were visualized with iTOL^49^. To calculate the microbiome-based leakage risk-score, the abundances of the significantly different genera in the preoperative sample were taken and normalized logarithmically. The score is the ratio between the multiplied genera abundances associated with sufficient anastomosis and the genera associated with leakage. This procedure is similar to the score used previously by Kartal et al^50^. With the calculated scores a receiver operating characteristic curves (ROC-curves) were performed in Prism 9.2 (GraphPad Software, Boston, USA). The same software was used to visualization of the data as well as perform Mann–Whitney U test and Friedman Test between a priori defined groups . The applied significands level nomenclature for all tests is: ns p > 0.05, * 0.049 > p > 0.01, ** 0.009 > p > 0.001, *** 0.0009 > p > 0.0001, **** p < 0.0001.

## Data availability

All data are included in the supplementary material.

### Supplementary Information

Supplementary Figures.

Supplementary Tables.
