# Supplementary material for: Prediction of anastomotic insufficiency based on the mucosal microbiome prior to colorectal surgery: a proof-of-principle study
Source: Sci Rep. 2024 Jul 3;14:15335. doi: 10.1038/s41598-024-65320-w (PMC11222535; doi:10.1038/s41598-024-65320-w)
Supplement: Supplementary file 1 — Supplementary Figures. [file 41598_2024_65320_MOESM1_ESM.pdf]

Supplementary Figures

Prediction of anastomotic insufficiency based on the mucosal microbiome prior to colorectal surgery.

Konrad Lehr[1], Undine Gabriele Lange[2], Noam Mathias Hipler[1], Ramiro Vilchez-Vargas[1], Albrecht Hoffmeister[3], Jürgen Feisthammel[3], Dorina Buchloh[4], Denny Schanze[5], Martin Zenker[5], Ines Gockel[2], Alexander Link[1]*, Boris Jansen-Winkeln[2,6]*

1 Department of Gastroenterology, Hepatology and Infectious Diseases,
Faculty of Medicine, Otto von Guericke University Magdeburg
2 Clinic and Polyclinic for Visceral, Transplant, Thoracic and Vascular Surgery,
Faculty of Medicine, University of Leipzig
3 Clinic and Polyclinic for Oncology, Gastroenterology, Hepatology and Pneumology,
Faculty of Medicine, University of Leipzig
4 Clinic for General and Visceral Surgery, Protestant Deaconess House Leipzig
5 Institute of Human Genetics, Faculty of Medicine,
Otto von Guericke University Magdeburg
6 Clinic for General, Visceral, Thoracic and Vascular Surgery, Clinic St. Georg Leipzig
* Authors contributed equally

CORRESPONDING AUTHORS
Boris Jansen-Winkeln (MD, Ph.D.)
Clinic and Polyclinic for Visceral, Transplant, Thoracic and Vascular Surgery
Faculty of Medicine, University of Leipzig
and
Clinic for General, Visceral, Thoracic and Vascular Surgery
Clinic St. Georg Leipzig
Phone: +49 341 9092200
Fax: +49 341 9092234
Email: boris.jansen-winkeln@sanktgeorg.de

Alexander Link (MD, Ph.D.)
Department of Gastroenterology, Hepatology and Infectious Diseases, Section of Molecular Gastroenterology and Microbiota-associated Diseases,
Otto-von-Guericke University, Magdeburg, Germany
Phone: +49 391 67 13100
Fax: +49 391 67 13105
Email: alexander.link@med.ovgu.de

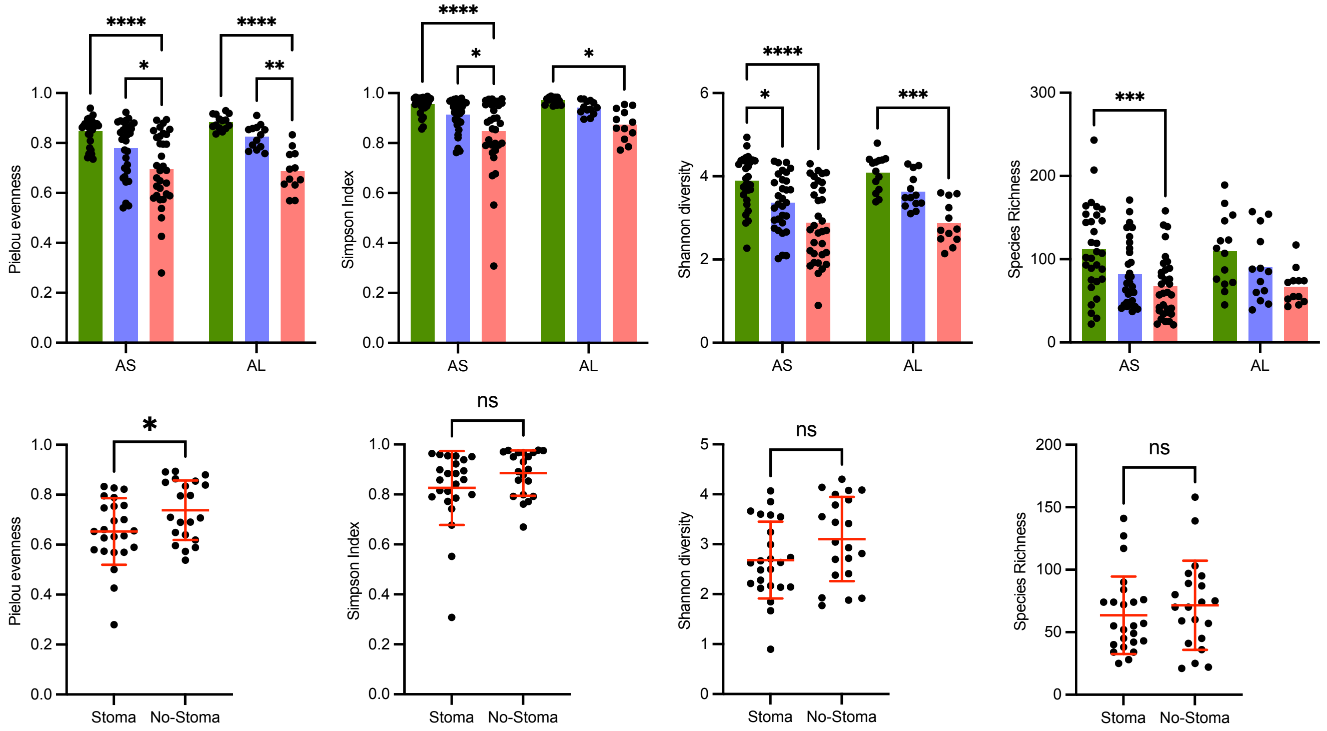

Supplementary Figure 1: Tow way comparison of diversity measurements between timepoints (green=PreSUR, blue=SUR, red=PostSUR) and the sufficient (AS) and leakage (AL) anastomoses sample type. Comparison of diversity measurements between patients with stoma and without after surgery.

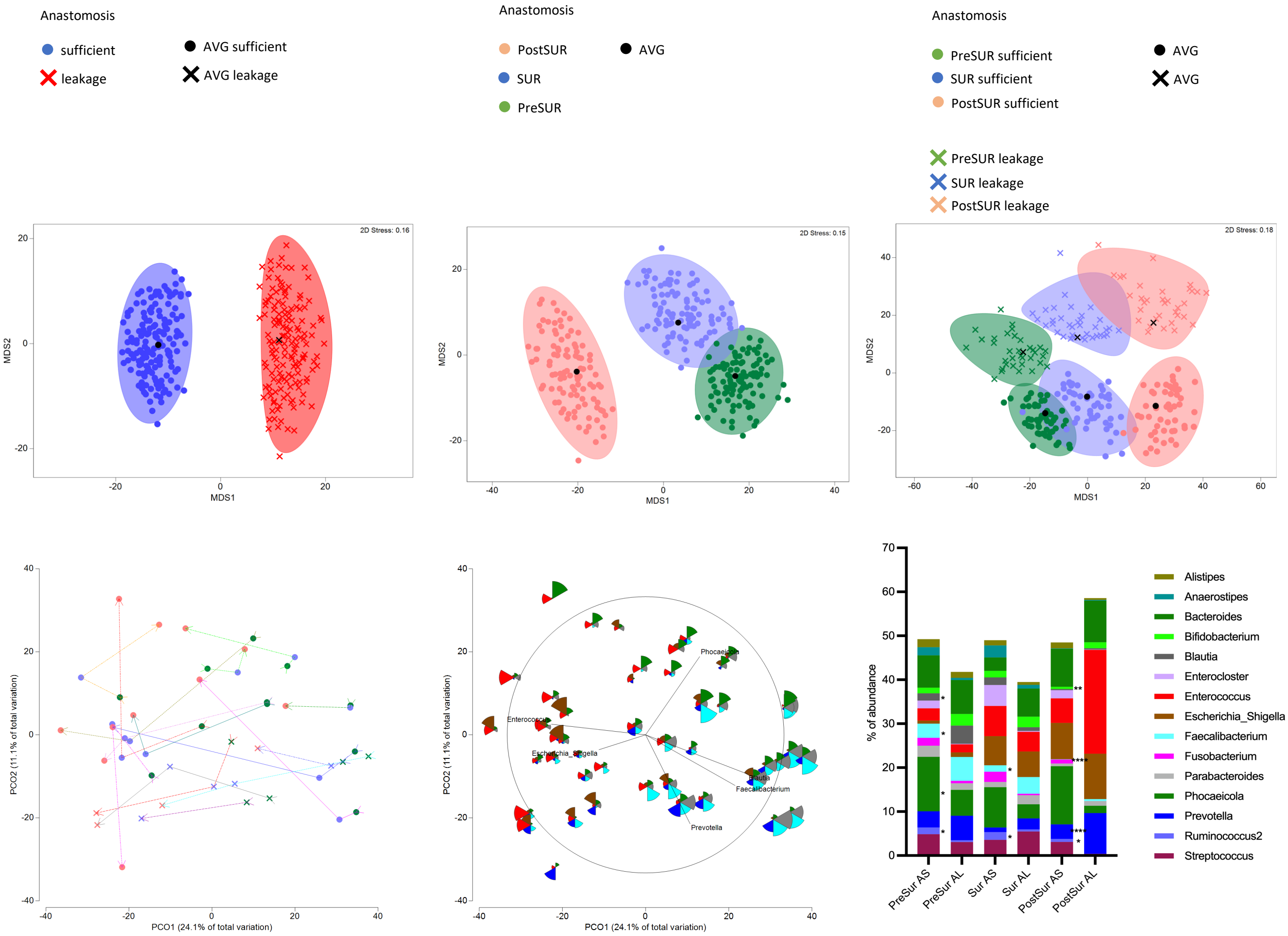

Supplementary Figure 2: Bootstrap average MDS for different groups of samples. PCO of distance among centroids underlying a Bray-Curtis resemblance measurement at genus level displaying groups with trajectories for each patient and with bubble-plot and vector overlay representing the abundance of genera across the sample. Comparison of the average relative abundance of bacteria in sufficient (AS) and leakage (AL) anastomoses sample for all timepoints separately.

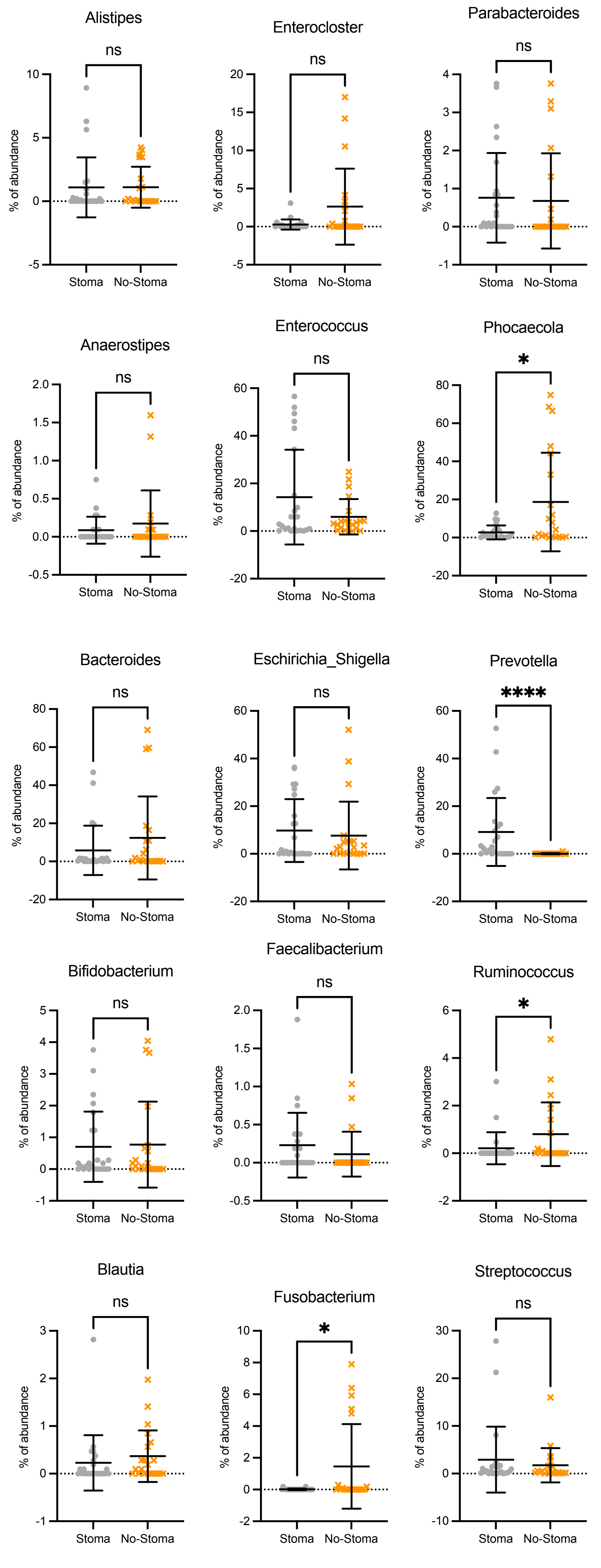

Supplementary Figure 3:

Comparison of different bacterial genera between patients with stoma and without after surgery.

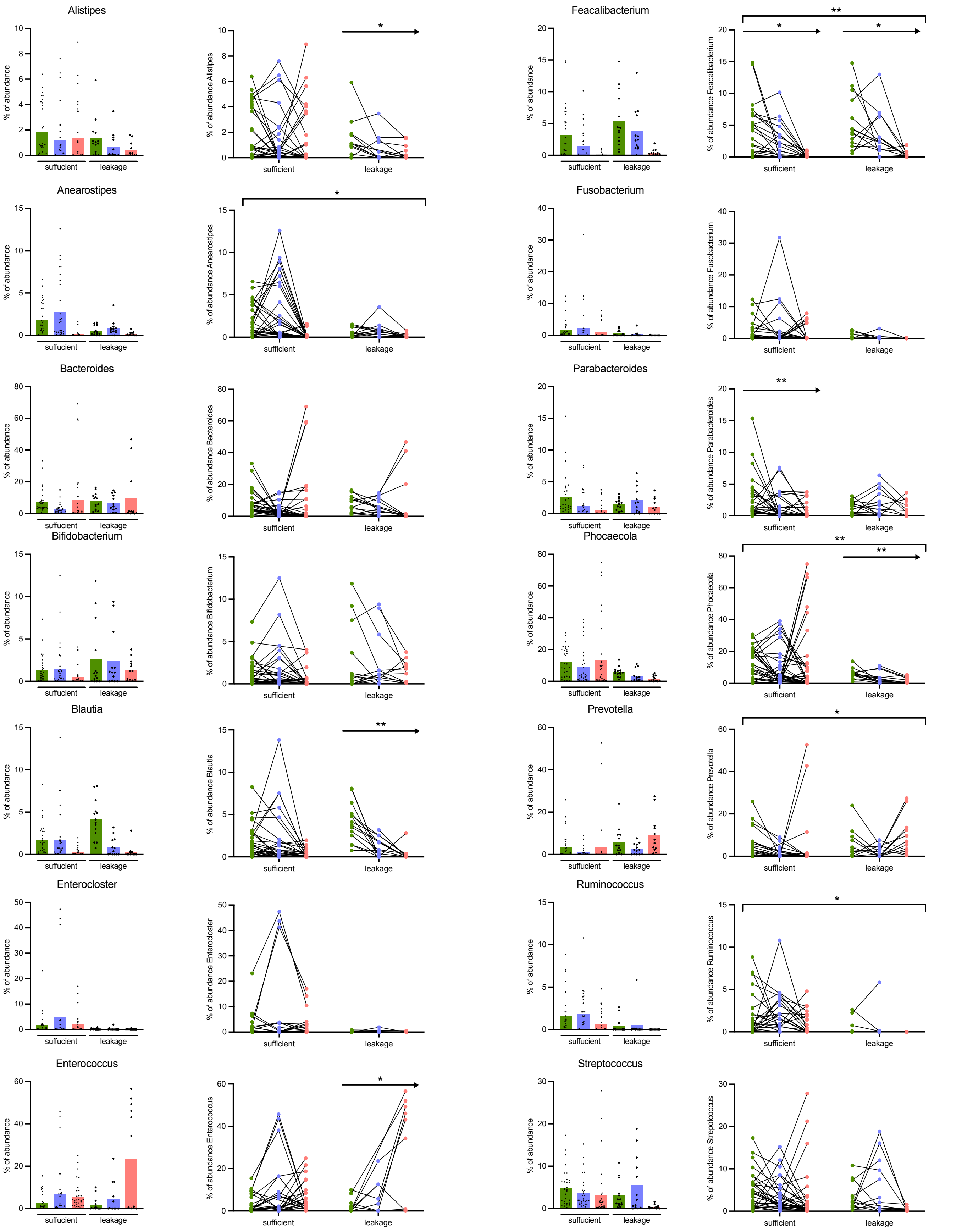

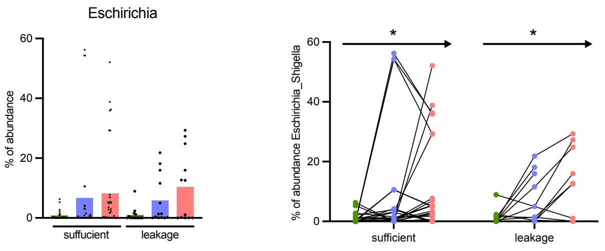

Supplementary Figure 4: Timepoint specific relative abundance of bacteria in sufficient and leakage anastomoses sample. Arrow displays statistically significant trend over time in one group (trendyspliner-Test). Bracket displays statistically significant differences between two groups over the full-time course (permuspliner-Test).

Supplementary Figure 5: Pathological characterisation of all patients.

Age		60 ± 8 years
Sex	male	12 (75 %)
	female	4 (25 %)
Tumour localisation	colon	4 (25 %)
	rectum	12 (75 %)
Anus praeter (stoma)	yes	9 (56 %)
	no	7 (44 %)
Anastomoses	sufficient	5 (31 %)
	leakage	11 (69 %)
Neoadjuvant radiochemotherapy	yes	8 (50 %)
	no	8 (50 %)
Histological classification	T 0-1	3 (19 %)
	T 2-3	13 (81 %)
	N 0	13 (81 %)
	N 1	3 (19 %)
	M 0	14 (87 %)
	M 1	2 (13 %)
Nicotine abuse	yes	3 (19 %)
	no	13 (81 %)
Diabetes mellitus Typ II	yes	6 (38 %)
	no	10 (62 %)
